# Hsp60 Post-translational Modifications: Functional and Pathological Consequences

**DOI:** 10.3389/fmolb.2020.00095

**Published:** 2020-06-04

**Authors:** Celeste Caruso Bavisotto, Giusi Alberti, Alessandra Maria Vitale, Letizia Paladino, Claudia Campanella, Francesca Rappa, Magdalena Gorska, Everly Conway de Macario, Francesco Cappello, Alberto J. L. Macario, Antonella Marino Gammazza

**Affiliations:** ^1^Section of Human Anatomy, Department of Biomedicine, Neuroscience and Advanced Diagnostic (BIND), University of Palermo, Palermo, Italy; ^2^Euro-Mediterranean Institute of Science and Technology (IEMEST), Palermo, Italy; ^3^Department of Medical Chemistry, Medical University of Gdańsk, Gdańsk, Poland; ^4^Department of Microbiology and Immunology, School of Medicine, University of Maryland at Baltimore-Institute of Marine and Environmental Technology (IMET), Baltimore, MD, United States

**Keywords:** Hsp60, chaperonin, canonical functions, non-canonical functions, post-translation modification, chaperonopathies

## Abstract

Hsp60 is a chaperone belonging to the Chaperonins of Group I and typically functions inside mitochondria in which, together with the co-chaperonin Hsp10, maintains protein homeostasis. In addition to this canonical role, Hsp60 plays many others beyond the mitochondria, for instance in the cytosol, plasma-cell membrane, extracellular space, and body fluids. These non-canonical functions include participation in inflammation, autoimmunity, carcinogenesis, cell replication, and other cellular events in health and disease. Thus, Hsp60 is a multifaceted molecule with a wide range of cellular and tissue locations and functions, which is noteworthy because there is only one *hsp60* gene. The question is by what mechanism this protein can become multifaceted. Likely, one factor contributing to this diversity is post-translational modification (PTM). The amino acid sequence of Hsp60 contains many potential phosphorylation sites, and other PTMs are possible such as O-GlcNAcylation, nitration, acetylation, S-nitrosylation, citrullination, oxidation, and ubiquitination. The effect of some of these PTMs on Hsp60 functions have been examined, for instance phosphorylation has been implicated in sperm capacitation, docking of H2B and microtubule-associated proteins, mitochondrial dysfunction, tumor invasiveness, and delay or facilitation of apoptosis. Nitration was found to affect the stability of the mitochondrial permeability transition pore, to inhibit folding ability, and to perturb insulin secretion. Hyperacetylation was associated with mitochondrial failure; S-nitrosylation has an impact on mitochondrial stability and endothelial integrity; citrullination can be pro-apoptotic; oxidation has a role in the response to cellular injury and in cell migration; and ubiquitination regulates interaction with the ubiquitin-proteasome system. Future research ought to determine which PTM causes which variations in the Hsp60 molecular properties and functions, and which of them are pathogenic, causing chaperonopathies. This is an important topic considering the number of acquired Hsp60 chaperonopathies already cataloged, many of which are serious diseases without efficacious treatment.

## Introduction

Post-translational modification (PTM) is a covalent change in an amino acid in a protein that can modify the properties and functions of the latter, for instance folding, ligand binding, migration to the place of residence, interaction with other molecules, and other specific roles, which in the case of molecular chaperones could be any of the various modes of chaperoning client polypeptides and any of their non-canonical tasks. The occurrence of a PTM depends on the spatial orientation of the target amino acid and on the neighboring residues in the protein molecule, which confer selectivity and reactivity of the former by affecting its electrophilic status (Santos and Lindner, 2017).

Hsp60 is a molecular chaperone that belongs to the chaperonins of Group I; it is named HSPD1 or Cpn60 in humans and is classically described as a mitochondrial resident that maintains protein homeostasis in the organelle. However, this chaperonin plays various other roles in health and disease, particularly as a pathogenic factor in a range of inherited and acquired chaperonopathies (Macario and Conway de Macario, 2005; Cappello et al., 2008, 2013, 2014; Marino Gammazza et al., 2017b; Hoter et al., 2019; van Eden et al., 2019). For these reasons, interest in Hsp60 has been steadily increasing in recent years, especially because it holds promise for developing new diagnostic and therapeutic procedures pertinent to common and serious chaperonopathies such as various types of cancer, and inflammatory and autoimmune disorders as well as for a range of neurodegenerative diseases (Macario and Conway de Macario, 2005; Cappello et al., 2008; Bross et al., 2012; Cappello et al., 2013, 2014; Marino Gammazza et al., 2016, 2017b; Campanella et al., 2018; Meng et al., 2018; Hoter et al., 2019; van Eden et al., 2019). For example, Hsp60 inhibitors and modulators are being actively evaluated as novel anti-cancer agents (Wang et al., 2013; Cappello et al., 2014; Meng et al., 2018; Stevens et al., 2019).

Hsp60 occurs not only inside mitochondria but also in other intracellular locations, for example the cytosol, and extracellularly, and its functions vary accordingly, depending on the interactors that surround it at the various locations. For example, inside mitochondria Hsp60 assists the folding and trafficking of other proteins, but in the cytosol it can favor apoptosis or the contrary, for example in some cancer cells, and can thus be anti- or pro-cancer, respectively (Campanella et al., 2014; Marino Gammazza et al., 2017b).

Structurally, the Hsp60 molecule has functional modules and three structural domains and if any of these modules-domains is altered by a PTM, its functions may be seriously impaired as shown, for example, with the chaperonin CCT (Macario and Conway de Macario, 2020). This type of modification might change Hsp60 from cytoprotective into pathogenic, causing a chaperonopathy. To the best of our knowledge, there is no article describing all known Hsp60 PTMs together, in a way that would be a useful resource for practitioners and scientists in their studies of Hsp60 chaperonopathies in patients or experimental models. The main goal of this article is to contribute to filling in this information gap. Consequently, we present a comprehensive review of known PTMs of Hsp60, with a brief discussion of the possible impact of a few of them on some of its properties and functions.

## Hsp60 Structure and Chaperoning Cycle

Hsp60 is highly conserved in evolution, from bacteria and archaea to complex plants and animals (Gupta, 1995; Marino Gammazza et al., 2012; Ansari and Mande, 2018).

**FIGURE 1:**
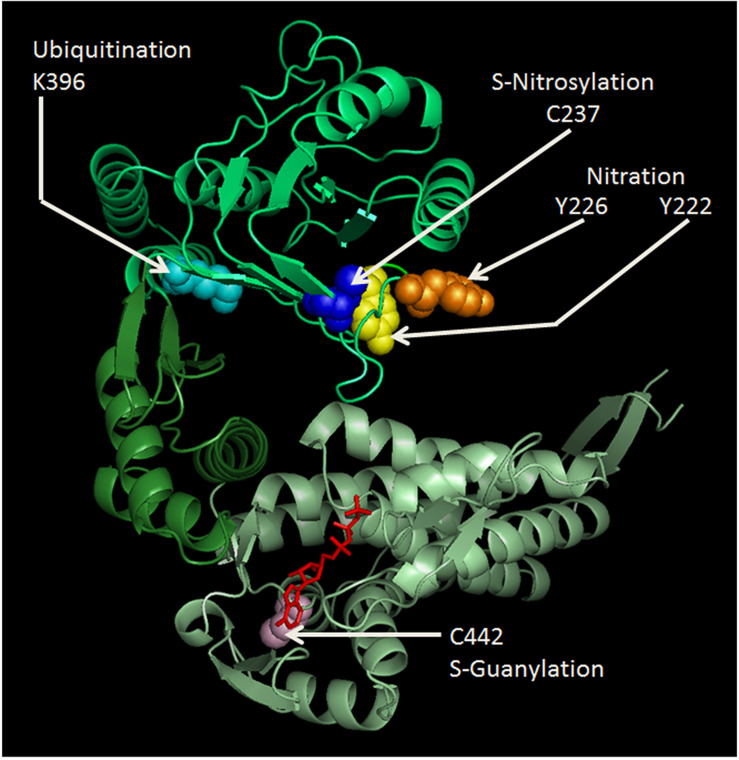
Cartoon representing the human Hsp60 monomer drawn to show some of the known PTM sites and their modifications. Amino acids shown are: Y222 (yellow), Y226 (orange), K396 (cyan), and C237 (blue) in the apical domain (lime); and C442 (light pink) and ATP (red) binding site in the equatorial domain (pale green). Nitration of the much conserved Y222 and Y226, and ubiquitination of K396 in the apical domain might seriously impair chaperoning functions, since this domain is crucial for Hsp10 and client protein binding. S-nitrosylation of C237 was found beneficial for the maintenance of mitochondrial DNA stability, during experimental peritonitis in mice (Suliman et al., 2010). C442 is located near the ATP-binding site in the equatorial domain and its S-guanylation might impair ATPase activity and oligomerization ability. The amino acid sequence of the human Hsp60 was retrieved from the PubMed website (http://www.ncbi.nlm.nih.gov/genbank/), using the accession number NM_002156. The cartoon was drawn using SWISS-MODEL (http://swissmodel.expasy.org/) accessible via the ExPASy web server (http://www.expasy.org/); and was visualized and modified by PyMol (http://www.pymol.org).

**FIGURE 2:**
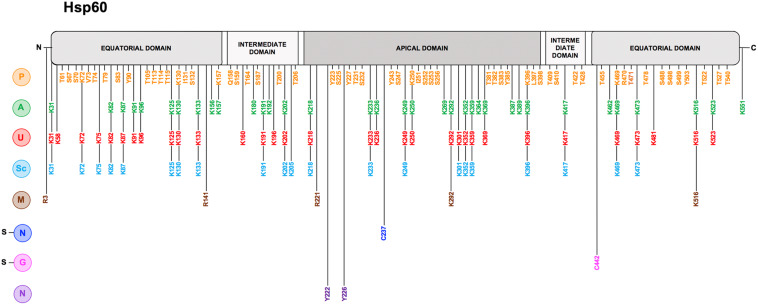
Hsp60 post-translational modifications. Linear representation of human Hsp60 with the N-terminal, 26 amino acids-long, mitochondrial import sequence (MIS) to the left; the two segments of the equatorial domain in gray (residues 30–157 and 434–548 in the Hsp60 full-length sequence), containing the ATP-binding pocket; the two segments of the intermediate domain in light gray (residues 158–214 and 402–433), connecting the equatorial and the apical domains; and the apical domain in dark gray (residues 215–401), involved in substrate-recruitment and co-chaperonin binding. On the left of the figure all reported PTMs are indicated with a letter with a color code: Phosphorylation (P) in orange, Acetylation (A) in green, Ubiquitination (U) in red, Succynilation (Sc) in light blue, Methylation (M) in brown, S-Nitrosylation (s-N) in blue, S-guanylation (s-G) in magenta, and Nitration (N) in dark purple. Along the linear representation of the Hsp60, aligned with each letter, the residues involved in the corresponding PTM are indicated with the same color as that of the pertinent modification. The data were obtained from the PTM database PhosphoSitePlus (http://www.phosphosite.org) and from the scientific literature.

In mammals, Hsp60 and its co-chaperone Hsp10 are classically located inside mitochondria where they constitute the protein folding apparatus with a mechanism elucidated using the bacterial homologues GroEL and GroES, respectively (references in Richardson et al., 1998; Cappello et al., 2014; Vilasi et al., 2018). Hsp60 forms a stable tetradecameric double-ring complex in the absence of Hsp10 and nucleotide (Enriquez et al., 2017). The crystal structure of Hsp60 in complex with Hsp10 shows a symmetric double-ring, American football-like structure with extensive interring contacts and the symmetry of the Hsp60 subunits within each ring observed in the bacterial chaperonin is not preserved in the human counterpart (Nisemblat et al., 2015). Moreover, the interring nucleotide asymmetry that characterizes the GroEL folding cycle is absent, because both Hsp60 rings are in the ADP-bound state. Hsp60 binds unfolded proteins catalyzing their folding in an ATP dependent manner (Weiss et al., 2016; Bhatt et al., 2018; Bigman and Horovitz, 2019). Hsp10 acts as a cap sitting on the outer border of the mouth of the heptameric ring, opening and closing the tetradecamer central cavity, regulating both the interactions of the Hsp60 monomers and ATP hydrolysis (Dubaquie et al., 1997; Richardson et al., 1998; Vilasi et al., 2018). Hsp60 monomers are formed of three structural domains named apical, intermediate and equatorial (Figures 1, 2): (i) the apical domain binds the substrate and the co-chaperone and it is implicated in ATP turnover; (ii) the intermediate domain connects the apical with the equatorial domain; and (iii) the equatorial domain facilitates interactions between the single subunits within a ring and between the two heptameric rings of the chaperonin (Braig et al., 1994; Ishida et al., 2018). Electron microscopic analysis of the human Hsp60 showed that the Hsp60/Hsp10 complex goes through a more complicated functional cycle than that of the GroEl/GroES complex, and this increased complexity depends on distinctive structural features of Hsp60 and of the Hsp60/Hsp10 complex. Hsp60 can start as a single ring that enters the double-ring cycle by binding to another ring along with Hsp10 and ATP. After ATP hydrolysis, Hsp60 releases ADP and Hsp10, returns to the single-ring structure and enters the next ATP-dependent cycle (Weiss et al., 2016; Enriquez et al., 2017; Bhatt et al., 2018; Bigman and Horovitz, 2019). Previous research had shown that mitochondrial Hsp60 exists in solution in dynamic equilibrium as monomer, heptamer (single ring), and tetradecamer (double ring), depending on protein concentration, temperature, and presence of cofactors (ATP and Hsp10) (Levy-Rimler et al., 2001). Also, biophysical methods have highlighted the importance of protein-protein interactions underlying the formation of stable Hsp60 oligomeric complexes (heptamers and tetradecamers), in equilibrium with minor populations of monomers, in aqueous solutions (Vilasi et al., 2014).

Data from the GroEL crystal structure and from the alignment of Hsp60 sequences from a wide range species have revealed highly conserved sequence segments and residues (Brocchieri and Karlin, 2000). The study of the connections of the conserved residues inside Hsp60 tri-dimensional structure and of their chemical and physical properties can lead to an understanding of the possible disruptive effects of PTMs on the protein stability and functions. One of the most complete papers regarding this topic reported several conserved residues between GroEL and Hsp60 (Brocchieri and Karlin, 2000). For example, 246-PLLIIAED-253 and 275-AVKAPGFGDRRK-286 are two conserved sequences of the apical domain containing five aliphatic residues and enriched in charged residues. The sequence 191-EGMQFDRGYISPY-203 between the intermediate and the apical domain contains several aromatic residues for substrate binding. As connection between the intermediate and the apical domains, the conserved segments 363-EKLQERLAKLAGGVAVIKVG-382 and 402-ATRAAVEEGIVPGGG-416 include charged residues at positions 275–286 and 363–382 and the glycine triplet at positions 402–416 represent the binding domain for ATP/ADP (Sigler et al., 1998). The apical domain contains also highly conserved hydrophobic/aromatic residues that contribute to substrate and co-chaperone binding such as Y199, Y203, Y222, F204, Y226, L234, L237, L259, V263, and V264 (Braig et al., 1994; Fenton et al., 1994). Any alteration, such as a PTM of the corresponding Hsp60 residues involved in substrate binding may result in functional defects, probably leading to protein misfolding and aggregation, and causing a chaperonopathy. The equatorial domain contains residues essential for the functioning of the chaperonin at positions 52–60 and 85–95 implicated in the binding of ATP/ADP and Mg^2+/^K^+^ ions (Brocchieri and Karlin, 2000). Any alteration of these sites or blocking them with a chemical compound might inactivate Hsp60. Other sites crucial for the typical chaperoning process mediated by GroEL/GroES during polypeptide folding are in the apical domain and represent the contact positions for GroES binding (L234, L237, and N265); these hydrophobic residues, highly conserved between species and necessary for substrate binding, contact GroES at the conserved hydrophobic residues I25, I26, L27, and A31 (Brocchieri and Karlin, 2000).

The formation and functioning of the Hsp60 tetradecamer depend on the inter-monomer and intra-ring connections. The interaction between equatorial domains of contiguous monomers involves the hydrophobic residues I6, L73, L513, T517, and V521 from one side and the residues V39, L40, I49, and I60 on the opposite surface. The connections are completed *via* the presence of opposite charged interactions between K4-E518 and E61-R36 (Brocchieri and Karlin, 2000). The conserved hydrophobic residue V464 represent the interactions between rings. The residues K105, E461, and E467, the residues A108, A109, and S463 with the opposite charged residues E434 and D345 contribute to the salt bridge K105-E434 and to allosteric switch (Chen et al., 1994; Brocchieri and Karlin, 2000; Sot et al., 2003). All these data regarding the chemical and physical characteristics of the residues distributed along GroEL and by similarity along Hsp60 domains show that several residues are crucial for the correct assembling of the two-ringed machine. Analysis of the crystal structure of the complex Hsp60/Hsp10 revealed some differences in the interring contact points of Hsp60 compared to GroEL but no differences were mentioned for other conserved and functionally important residues (Nisemblat et al., 2015). The symmetric key of A109 in GroEL is replaced with a salt bridge between K109 and E105 in Hsp60 and a new symmetric hydrophobic interaction is formed between two A10 as well as a new symmetric hydrogen bond is formed between two D11. Moreover, the salt bridge between E461 and R452 that is present in GroEL is replaced by a salt bridge between E462 and K449 in Hsp60 (Nisemblat et al., 2015).

PTM of these and other residues, will most likely cause a failure of tetradecamer formation, impairing Hsp60 chaperoning ability and causing disease, a chaperonopathy.

## Hsp60 Post-Translational Modifications

Hsp60 is a multifaceted molecule with canonical and non-canonical functions in a variety of physiological and pathological processes depending among other factors on cellular localization, Table 1. Any of the Hsp60 function may be affected by PTMs. It is, therefore, necessary to survey some of the roles of Hsp60 to gain insights on where, when, and how a PTM can make a significant impact.

**TABLE 1:** Hsp60 localization and functions.

**Localization**	**Functions**	**References**
Mitochondria	Replication and transmission of mitochondrial DNA	Kaufman et al., 2000, 2003
	Protein folding	Weiss et al., 2016
	Pro-survival or pro-death functions	Itoh et al., 2002; Chandra et al., 2007
Cytosol	Pro-survival and/or pro-death functions	Itoh et al., 2002; Chandra et al., 2007; Campanella et al., 2008; Chun et al., 2010; Caruso Bavisotto et al., 2017b; Zhou et al., 2018
	Activation of the apoptotic cascade	Gupta and Knowlton, 2005
Cell membrane	Membrane transport, cell–cell signaling	Belles et al., 1999; Dziewanowska et al., 2000; Pfister et al., 2005; Cappello et al., 2008; Merendino et al., 2010; Campanella et al., 2012; Caruso Bavisotto et al., 2017a
	Immune system alerting	Habich and Burkart, 2007; Cappello et al., 2009; Marino Gammazza et al., 2017b
Extracellular space	Either pro- or anti-inflammatory	Watanabe et al., 2003; Swaroop et al., 2016, 2018
	Pro-tumoral signal	Campanella et al., 2015a; Caruso Bavisotto et al., 2017b; Marino Gammazza et al., 2017a
	Immune system alerting	Xiao et al., 2005; Habich and Burkart, 2007; Shamaei-Tousi et al., 2007; Marino Gammazza et al., 2014
	Correlation with disease severity and cardiovascular risk	Bonanad et al., 2013
Extracellular vesicles	Activation of late apoptosis in cardiomyocyte	Gupta and Knowlton, 2007; Malik et al., 2013
	Tumor progression signal	Campanella et al., 2015a, b; Caruso Bavisotto et al., 2017a; Marino Gammazza et al., 2017a; Campanella et al., 2019

In humans, Hsp60 is encoded by a nuclear gene (*HSPD1*) on chromosome 2q33.1, and subsequently translated in the cytosol (Jindal et al., 1989). The protein consists of 573 amino acids (Figure 2), including a mitochondrial import signal (MIS) at the N-terminus of 26 amino acids necessary for its import into mitochondria (Singh et al., 1990) and, in addition, a series of G repeats at the C-terminus with unknown function (Brocchieri and Karlin, 2000). The mitochondrial import mechanism of Hsp60 is very complex involving the potential of mitochondrial membranes (Yogev and Pines, 2011) and other chaperones present in the matrix space (e.g., Hsp70) and in the cytosol (Singh et al., 1990; Vilasi et al., 2018). Intra-mitochondrial cleavage of the MIS generates the mature Hsp60 protein, with 547 amino acids and a molecular mass of about 60 kDa.

Proteins with PTM are involved in all fundamental cellular processes. For example, lysine modification of nuclear proteins play a crucial role in gene regulation (McIntyre and Woodgate, 2015), and modified proteins are critical to maintain protein homeostasis (Minguez et al., 2012; Lindstedt et al., 2019). Likewise, phosphorylation, S-nitrosylation, and acetylation of mitochondrial proteins occur to modulate their functions inside the organelle (Foster et al., 2009; Mailloux et al., 2014).

PTMs can drastically change the function of a protein, which makes the understanding of the networks in which the modified protein is involved very difficult. There is abundant information regarding the effects of PTMs on some molecular chaperones like Hsp70 and Hsp90 (Cloutier and Coulombe, 2013), but comparatively little is known about Hsp60 PTMs. Given the key role of Hsp60 in the regulation of cellular homeostasis, the decoding of the different PTMs that affect it can represent a turning point in many areas of cellular research. Some PTMs occurring in a specific and sequential manner describe a sort of code, the interpretation of which could reveal much about the activity of molecular chaperones in cells (Cloutier and Coulombe, 2013).

Hsp60 PTMs have not been investigated extensively even though elucidation of the impact of modifications of this multifaceted molecule will most likely shed light on the various mechanisms underpinning the diverse roles and migration of the chaperonin. It is possible that PTMs would affect key Hsp60 properties and functions if the modifications occur at one or more of the various critical sites on the Hsp60 molecule, such as the ATP-binding and substrate binding sites, the sites involved in intra- and inter-ring contacts, and sites pertaining to networking and to migrating and taking residence in the different intra- and extra-cellular locations in which Hsp60 resides and works. In this section we discuss Hsp60 PTMs described in the literature and the effects of these modifications.

### Phosphorylation

Among the various PTMs that can occur on Hsp60 (Table 2 and Figure 2), phosphorylation is involved in physiological and pathological processes. The amino acid sequence of Hsp60 contains a number of potential phosphorylation sites (K72-V73-T74, K130-I131-S132, K157-Q158-S159, K250-I251-S252, K396-L397-S398, and K469-R470-T471) and the potential impact of their modification is still unclear (Jindal et al., 1989; Khan et al., 1998). Hsp60 can be tyrosine phosphorylated at Y227 and Y243 (Rikova et al., 2007; Gu et al., 2011) and Hsp60 tyrosine phosphorylation is required for its surface activation (Asquith et al., 2004). Under physiological conditions, for instance during the sperm-zona recognition, Hsp60 tyrosine phosphorylation triggers conformational changes, contributing to the activation of the zona pellucida receptor complex on the surface of mammalian spermatozoa and, thus, leads to sperm capacitation (Asquith et al., 2004). In an *in vitro* model of leukemia, the extra-mitochondrial form of Hsp60 localized in the plasma-cell membrane was found to interact with the histone 2B (H2B) and its phosphorylation regulated the docking of H2B by Hsp60 (Khan et al., 1998). Differential phosphorylation patterns of Hsp60 have been observed in rat hepatomas, in which the phosphorylation regulates the functions of microtubule associated proteins (Albrethsen et al., 2011). Phosphorylated Hsp60 was identified as a molecular mediator for α3β1 integrin activation in the adhesion of metastatic breast cancer cells to the lymph nodes and to bone osteoblasts (Barazi et al., 2002). Many malignant cells require tyrosine phosphorylation of Hsp60 to escape immunosurveillance by NK and CD8 T cells (Leung et al., 2015). Hyperglycemia induces an increased phosphorylation pattern of Hsp60, which might be associated to mitochondrial dysfunction (Gu et al., 2011). In response to rotavirus infection, phosphorylation and the subsequent transient degradation of mitochondrial Hsp60 are associated with an escape mechanism by which the virus leads to a delay of the early apoptosis activation (Chattopadhyay et al., 2017).

**TABLE 2:** Examples of Hsp60 PTM.

**PTM^a^**	**Modified amino acid or site**	**Effect/function affected**	**References**
Phosphorylation	Tyrosine	Sperm capacitation	Asquith et al., 2004
	Serine/threonine	Docking of H2B and microtubule-associated proteins	Khan et al., 1998; Albrethsen et al., 2011
	Serine/threonine	Mitochondrial dysfunction	Gu et al., 2011
	Not defined	Tumor invasiveness	Barazi et al., 2002
	Tyrosine	Immune escape	Leung et al., 2015
	Tyrosine at positions 90, 223, 227, and 503	Delay of apoptosis activation	Chattopadhyay et al., 2017
O-GlcNAcylation, N-glycosylation	Serine and/or threonine	Pro-apoptotic	Kim et al., 2006; Gu et al., 2011; Gorska et al., 2013; Marino Gammazza et al., 2017a
	Lysine	Modulation of Hsp60/Hsp10 complex activity	Lu et al., 2015; Bross and Fernandez-Guerra, 2016
	N-linked glycosylation sites (N103, N230 and N426)	Immune system modulation	Helenius and Aebi, 2001; Barazi et al., 2002; Hayoun et al., 2012
Nitration	Cysteine 442	Stability of the mitochondrial permeability transition pore	Ghosh et al., 2010; Rahaman et al., 2014
	Tyrosine 222, and 226	Inhibition of Hsp60 folding activity	Campanella et al., 2015a
	Hsp60 ATP binding site (amino acid not defined)	Disturbance of insulin secretion	Koeck et al., 2009
S-nitrosylation	Cysteine	Cardioprotective effects	Sun et al., 2007; Lin et al., 2009; Kohr et al., 2014
	Cysteine 237	Mitochondrial stability and endothelial integrity	Suliman et al., 2010; Huang et al., 2012
Citrullination	Not defined	Pro-apoptotic	Lu et al., 2016
Methylation	Lysine 490; Arginine	Pro-proliferative	Lim et al., 2008; Lim et al., 2010; Cao et al., 2013
Oxidation	Not defined	Response to cellular injury and cell migration	Suh et al., 2004; Lin et al., 2016
Biotinylation	Lysine	Anti-oxidant effect	Li et al., 2014
Ubiquitination	Lysine 396	Regulation of stress-activated ubiquitin-proteasome pathway	Leach et al., 2011; Tang et al., 2013; Marino Gammazza et al., 2017a

*^*a*^PTM, post-translation modification.*

### O-GlcNAcylation, N-Glycosylation, and Acetylation

The O-linked-b-N-acetylglucosamine modification (O-GlcNAcylation) of Hsp60 occurs at the serine and/or threonine residues, which is important for regulating a range of biological activities of Hsp60, including metabolism, signaling, and transcription (Gu et al., 2011; Gorska et al., 2013; Marino Gammazza et al., 2017a). Under high glucose condition, also an aberrant O-GlcNAcylation occurs in Hsp60 of myoblasts that it is associated with its phosphorylation, creating a crosstalk related to mitochondrial metabolism (Gu et al., 2011). In pancreatic β-cells, the O-GlcNAcylation of Hsp60 inhibits its binding to Bax, which is a pro-apoptotic protein that becomes free to translocate to mitochondria and activate cell death (Kim et al., 2006).

In tumors, as well as in normal cells under stress, N-glycosylated Hsp60 is expressed on the cell surface or secreted extracellularly (Barazi et al., 2002). The chaperonin has three potential N-linked glycosylation sites, N103, N230, and N426 (Helenius and Aebi, 2001). On the surface of a tumor, N-glycosylated Hsp60 would be able to modulate the immune response within the tumor microenvironment (Hayoun et al., 2012).

Our group demonstrated that Hsp60 hyperacetylation, following anticancer treatment in human tumor cells, contributes to the death of these cells (Gorska et al., 2013). The post-translational hyperacetylation of Hsp60 might affect its interaction with p53 and signal for Hsp60 degradation via the ubiquitin-proteasome system, thus leading to cellular senescence and tumor growth arrest (Marino Gammazza et al., 2017a).

Large-scale proteomic approaches showed numerous mitochondrial acetylated proteins; however, in most cases, their regulation by acetyltransferases and deacetylases remains unclear. Sirtuin3 (SIRT3) is an NAD+-dependent mitochondrial protein deacetylase that regulates enzymes in crucial metabolic pathways (Rardin et al., 2013). SIRT3-dependent acetylation of the Hsp60 co-chaperone, Hsp10 (Lys-56 residue) is critical in the dynamic interaction between the Hsp60/Hsp10, affecting protein folding in the mitochondria (Lu et al., 2015). Lysine acetylation is key for the Hsp60/Hsp10 complex activity. Therefore, alteration of the acetylation levels in certain amino acids of Hsp60 can promote development of disease (Bross and Fernandez-Guerra, 2016).

### Nitration and S-Nitrosylation

Mitochondrial metabolism and integrity are ensured by the correct functioning of mitochondrial proteins, including their adequate response to stress. A particular PTM, related to nitration, i.e., S-guanylation, was identified in the Hsp60 C442, which is located near the ATP-binding site and can play a crucial role in its chaperoning activity and in the ability to oligomerize (Rahaman et al., 2014; Figure 1). This modification may influence Hsp60 stability and the functioning of the mitochondrial chaperoning subsystem with regard to the opening of mitochondrial permeability transition pore (Ghosh et al., 2010; Rahaman et al., 2014). Nitric oxide (NO) induces S-nitrosylation of Hsp60 C237 (Figure 1), facilitating interactions with the proteins required to maintain mitochondrial DNA stability during experimental *E. coli* peritonitis in mice (Suliman et al., 2010). Also, Hsp60 S-nitrosylation might mediate the beneficial effect of statins on endothelial integrity, but the mechanism remains to be explained (Huang et al., 2012). The positive effect of S-nitrosylation on proteins, included Hsp60, may be of significance in the regulation of energy production in mitochondria and, thereby, would play a role in cytoprotection, as investigated in cardiac injury *in vivo* models, in which a pathway involving the S-nitrosylation of key cardioprotective proteins was described (Sun et al., 2007; Lin et al., 2009). Along this line of thought, a role of GAPDH as mediator of NO transport in mitochondria has been proposed (Kohr et al., 2014). Among the cysteine residues involved in S-nitrosylation, C442 and C237 are present in Hsp60 (Figure 1) but not in GroEL and represent interesting sites for the development of electrophilic Hsp60-binding compounds (Cappello et al., 2014).

Hsp60 nitration, e.g., in response to an excess of ROS, was shown to decrease ATP-hydrolysis activity, which disrupts the interaction of the chaperonin with its substrates and, thus, inhibits its substrate-folding ability (Campanella et al., 2015a). These serious effects of nitration happen because the modification most probably occurs in the highly conserved residues Y222 and Y226 of the apical domain (Figure 1), and this domain is crucial for Hsp10 and substrate binding by Hsp60. In pancreatic β-cells, Hsp60 nitration on the ATP binding site affects the process by which the insulin is secreted in secretory granules (Koeck et al., 2009). Therefore, this could be a mechanism underlying the onset and progression of diabetes (Koeck et al., 2009).

Hsp60 nitration could be a signal to release it into the extracellular space and circulation, for example via exosomes, where it would interact with the immune system (Caruso Bavisotto et al., 2013, 2017a; Campanella et al., 2014, 2015a).

In mitochondria, NO has an ambiguous role. On the one hand, NO produces various inhibiting effects on electron transport, and prolonged exposure is pro-apoptotic (Campanella et al., 2015a). On the other hand, NO induces S-nitrosylation of Hsp60 C237 (Figure 1), facilitating interactions with the proteins required to maintain mitochondrial DNA stability during experimental *E. coli* peritonitis in mice (Suliman et al., 2010). Also, Hsp60 S-nitrosylation could mediate the beneficial effect of statins on endothelial integrity, but the mechanism remains unclear (Huang et al., 2012).

### Citrullination and Methylation

Hsp60 is also known to be subjected to citrullination or deamination, which is a conversion of the amino acid arginine into the amino acid citrulline (Jiang et al., 2013). Citrullinated Hsp60 was found in the surface of cells of a human sarcoma osteogenic cell line, inducing apoptosis through TLR4 signaling, a mechanism involved in joint damage in patients with rheumatoid arthritis (Lu et al., 2016). Data from different cell lines demonstrated that one functional methylation present on Hsp60 is the mono-methylated lysine 490 (K490me1) (Cao et al., 2013). Senescent fibroblasts showed low level of asymmetric arginine di-methylation of Hsp60 compared to low-passage fibroblasts. This means that arginine asymmetric di-methylation of Hsp60 is correlated with the proliferation potential of cells and might be useful as a marker of cellular senescence (Lim et al., 2008, 2010).

### Oxidation and Biotinylation

As a redox sensitive protein, Hsp60 is oxidized in HepG2 cells exposed to alcohol (Suh et al., 2004) and it is responsible for cellular injury and cell migration (Lin et al., 2016). The C-terminal motif in Hsp60 might be considered a ROS acceptor thanks to a combination of PTMs in its residues (Li et al., 2014). It has been proposed that biotinylation of lysines in Hsp60 close proximity to sulfoxidation sites (methionine) contributes toward the elimination of ROS via the methionine/methionine sulfoxide reductase pathway in human cell cultures (Li et al., 2014).

### Ubiquitination

In monocytes treated with azacytidine, a stress response occurs with Hsp60 upregulation and ubiquitination in its K396 residue (Tang et al., 2013; Figure 1). The role of this PTM is still unclear, but it may play important roles in key cellular processes, such as in the stress-activated ubiquitin-proteasome pathway (Tang et al., 2013; Marino Gammazza et al., 2017a). A mutation in the target point of the small ubiquitin-like modifier (SUMO) contributes to aberrant growth morphology in *Candida albicans*, confirming the importance of Hsp60 for cell survival under certain stress conditions (Leach et al., 2011).

## Conclusion and Perspectives for the Future

PTMs of Hsp60 have effects on its properties and functions, for instance ATP and substrate binding, and interaction with the co-chaperonin Hsp10, all of which in turn very likely have an impact on the chaperoning ability and on any of the other roles played by this chaperonin. Sites that undergo PTM are distributed in all structural domains of Hsp60 and can affect any of its functional modules, suggesting that any one of the many functions, canonical and non-canonical, of this chaperonin may be affected by the modifications. Since Hsp60 is essential to the maintenance of cellular and tissue physiology, it is of great interest to elucidate which PTMs occur in health and in the various diseases, i.e., Hsp60 chaperonopathies, in which the chaperonin is known or suspected to play an etiopathogenic role. Likewise, it would be very useful to identify PTMs that control, or at least partly determine, the Hsp60 locale of residence inside and outside cells. The chaperonin may act intracellularly or at sites distant from its cell of origin, and the destination may be dictated by specific modifications. This emphasizes the need for more studies on Hsp60 PTMs, particularly in cancer and other serious diseases, in which spread of the disease may be associated with Hsp60 migration or with other aberrant properties of the chaperonin that make it pathogenic. Furthermore, learning about PTMs and their effects on the properties and functions of Hsp60 will reveal clues on what sites and modifications may be used to either block the chaperonin (negative chaperonotherapy in case Hsp60 is an etiopathogenic factor), or to boost its activity (positive chaperonotherapy in cases of chaperonopathies by defect). In this regard, the newly reported crystal structure of the Hsp60/Hsp10 complex (Gomez-Llorente et al., 2020) will be instrumental to dissect the possible effects of PTMs on structure and function.

## Author Contributions

AM, EC, FC, and AJM conceived the idea and performed the final editing and revision. CCB, GA, LP, and AM collected material, wrote, and revised the manuscript. AV prepared the figures. CC, FR, and MG reviewed the conclusions. All the authors read and approved the manuscript.

## Conflict of Interest

The authors declare that the research was conducted in the absence of any commercial or financial relationships that could be construed as a potential conflict of interest.
